# Colo‐Protective Effects of Pentoxifylline Alone or in Combination With Mesalamine in Colitis Through Sphingosine Kinase 1/Sphingosine 1 Phosphate, and Zonula Occuldin 1 Pathways: New Molecular Approach

**DOI:** 10.1002/prp2.70115

**Published:** 2025-05-19

**Authors:** Fatemah A. Alherz, Mahmoud S. Abdallah, Esraa M. Mosalam, Mostafa M. Bahaa, Thanaa A. Elmasry, Mohamad A. El‐Gammal, Walaa A. Negm, AyaIbrahim Elberri, Nora Elshorbagi, Hend E. Abo Mansour, Amir O. Hamouda, Muhammed M. Salahuddin, Mohamed Yasser, Mamdouh Eldesoqui, Sarah Alrubia, Amsha S. Alsegiani, Eman El‐Khateeb, Mohamed Kh. ElMahdy, Eman Wahsh

**Affiliations:** ^1^ Department of Pharmaceutical Sciences, College of Pharmacy Princess Nourah Bint Abdulrahman University Riyadh Saudi Arabia; ^2^ Department of PharmD, Faculty of Pharmacy Jadara University Irbid Jordan; ^3^ Biochemistry Department, Faculty of Pharmacy Menoufia University Shebin EL‐Kom Menoufia Egypt; ^4^ Pharmacy Practice Department, Faculty of Pharmacy Horus University New Damietta Egypt; ^5^ Pharmacology and Toxicology Department, Faculty of Pharmacy Tanta University Tanta Al‐Gharbia Egypt; ^6^ Pharmacologyand Toxicology Department, Faculty of Pharmacy Sinai University, Arish Branch Arish Egypt; ^7^ Pharmacy Practice Department, Faculty of Pharmacy Sinai University, Arish Branch Arish Egypt; ^8^ Department of Pharmacology and Biochemistry, Faculty of Pharmacy Horus University New Damietta Egypt; ^9^ Department of Pharmacology and Toxicology, Faculty of Pharmacy Mansoura National University Gamasa Mansoura Egypt; ^10^ Pharmacognosy Department, Faculty of Pharmacy Tanta University Tanta Al‐Gharbia Egypt; ^11^ Genetic Engineering and Molecular Biology Division, Department of Zoology, Faculty of Science Menoufia University Shebin El‐Kom Menoufia Egypt; ^12^ Pharmacology and Toxicology Department, Faculty of Pharmacy Sinai University, East Kantara Branch New City El Ismailia Egypt; ^13^ Department of Pharmaceutics, Faculty of Pharmacy Port Said University Port Said Egypt; ^14^ Department of Pharmaceutics, Faculty of Pharmacy East Port Said National University Port Said Egypt; ^15^ Department of Pharmaceutics and Industrial Pharmacy, Faculty of Pharmacy Horus University New Damietta Egypt; ^16^ Department of Basic Medical Sciences, College of Medicine AlMaarefa University Riyadh Saudi Arabia; ^17^ Pharmaceutical Chemistry Department, College of Pharmacy King Saud University Riyadh Saudi Arabia; ^18^ Clinical Pharmacy Department, Faculty of Pharmacy Tanta University Tanta Al‐Gharbia Egypt

**Keywords:** IL6/STAT3, Nrf2/HO‐1, PTX, SPHK/S1P, ulcerative colitis, ZO‐1

## Abstract

Multiple signaling pathways have been implicated in the pathogenesis of ulcerative colitis (UC), including Sphingosine Kinase 1 (SPHK)/Sphingosine‐1‐Phosphate (S1P), AMP‐activated protein kinase (AMPK)/mammalian target of rapamycin (mTOR)/NLR family pyrin domain‐containing 3 (NLRP3), zonula occludens‐1 (ZO‐1), and signal transducer and activator of transcription 3 (STAT3). We aimed to investigate the Colo protective and anti‐ulcerative effects of pentoxifylline (PTX) in a rat model of UC. Colitis was induced by intracolonic administration of 2 mL of 3% (v/v) acetic acid (AA). Thirty‐five rats were randomly assigned to five groups (*n* = 7 each): normal control, colitis, mesalamine, PTX, and a combination of PTX plus mesalamine. Disease activity was assessed using the disease activity index, colon weight and length measurements, histological examination, and immunohistochemical detection of caspase‐3. Colonic tissue homogenates were analyzed for interleukin‐6 (IL‐6), S1P, SPHK, mTOR, heme oxygenase‐1 (HO‐1), nuclear factor erythroid 2–related factor 2 (Nrf2), AMPK, and STAT3 levels. Gene expression of ZO‐1 and NLRP3 was also evaluated. Intracolonic AA induced marked functional, biochemical, and inflammatory damage to colonic tissue. Treatment with PTX, mesalamine, or their combination significantly attenuated these effects. Specifically, all treatments reduced levels of IL‐6, S1P, SPHK, mTOR, STAT3, NLRP3, and caspase‐3, while increasing levels of ZO‐1, HO‐1, Nrf2, and AMPK. The combination treatment group exhibited near‐complete restoration of normal colonic architecture, characterized by intact crypt morphology and minimal fibrosis in the lamina propria. PTX attenuated inflammation, apoptosis, and oxidative stress in colitis, supporting its potential as an adjuvant therapy in UC management.

## Graphical abstract

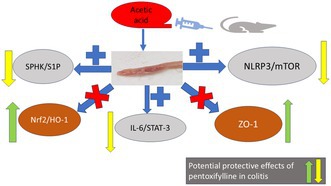

**Abbreviations**
AA: acetic acid
AIM2: absent‐in‐melanoma 2
AMPK: adenosine monophosphate‐activated protein kinase
ANOVA: one‐way analysis of variance
ARE: antioxidant response element
cAMP: cyclic adenosine monophosphate
DAI: the disease activity index
GAPDH: glyceraldehyde 3‐phosphate dehydrogenase
H and E: hematoxylin and eosin
HO‐1: heme oxygenase
IBD: inflammatory bowel disorders
IL‐6: interleukin 6
IL‐6R: IL‐6 receptor
LRR: pyrin and leucine‐rich repeat
MAPK: mitogen‐activated protein kinase
mTOR: mammalian target of rapamycin
mTORC1: mTOR complex 1
NF‐κB: Nuclear factor kappa B
NLRP3: NLR family pyrin domain containing 3
NOD: nucleotide‐binding oligomerization domain
NOS: nitric oxide synthase
Nrf2: nuclear factor erythroid‐2‐related factor 2
PKA: protein kinase A
PRR: pattern recognition receptor
PTX: pentoxifylline
RT‐PCR: reverse transcription polymerase chain reaction
S1P: Sphingosine 1 phosphate
SPHK: sphingosine kinase
STAT3: signal transducer and activator of transcription 3
TGF‐β: tumor growth factor‐beta
TNF‐α: tumor necrosis factor‐alpha
TSC2: tuberous sclerosis protein 2
UC: ulcerative colitis
ZO‐1: zonula occludin 1

## 1 Introduction

Ulcerative colitis (UC) is a type of inflammatory bowel disease (IBD) characterized by chronic inflammation and destruction of the colonic and rectal mucosa. Common symptoms include abdominal pain, cramps, rectal pain, bloody diarrhea, weight loss, fever, the presence of pus in the stool, nausea, vomiting, mouth ulcers, arthritis, and delayed growth in children [1]. A major contributor to UC pathogenesis is the imbalance between proinflammatory and anti‐inflammatory mediators [2].

Interleukin‐6 (IL‐6) is a pleiotropic cytokine implicated in the development of various inflammatory disorders, including IBD [3]. In patients with UC, the IL‐6 trans‐signaling pathway is initiated by the interaction of IL‐6 with its receptor (IL‐6R), followed by recruitment of the glycoprotein 130 (gp130) co‐receptor [4]. This complex activates Janus kinases (JAKs) and the downstream signal transducer and activator of transcription 3 (STAT3) [5]. Disruption of IL‐6‐mediated STAT3 activation has been shown to attenuate colitis severity in animal models, highlighting the central role of IL‐6/STAT3 signaling in UC progression [6].

Tumor necrosis factor‐alpha (TNF‐α), a key proinflammatory cytokine produced by colonic macrophages, plays a pivotal role in UC by activating sphingosine kinase 1 (SPHK1), which catalyzes the production of sphingosine‐1‐phosphate (S1P) [7]. In response to TNF‐α, SPHK1 activity in the colonic mucosa upregulates adhesion molecules and induces nitric oxide (NO) production via nitric oxide synthase (NOS), promoting macrophage and neutrophil infiltration. This cascade generates reactive oxygen species (ROS) that exacerbate mucosal inflammation and tissue injury [8].

The nuclear factor erythroid 2–related factor 2 (Nrf2) is a crucial transcription factor that regulates antioxidant defense mechanisms [9]. Under homeostatic conditions, Nrf2 is sequestered in the cytosol by Kelch‐like ECH‐associated protein 1 (Keap1). Upon oxidative stress, Nrf2 translocates to the nucleus, binds to the antioxidant response element (ARE), and activates the expression of phase II detoxifying enzymes, such as heme oxygenase‐1 (HO‐1), which are essential for cellular protection [10].

Adenosine monophosphate‐activated protein kinase (AMPK) is a conserved cellular energy sensor that regulates metabolic homeostasis by enhancing fatty acid and glucose uptake and promoting their oxidation [11]. AMPK activation also counteracts a variety of pathological conditions, including inflammation, insulin resistance, and ectopic fat deposition [12]. The mammalian target of rapamycin (mTOR), a serine/threonine kinase, integrates nutrient and growth signals to regulate cell growth and proliferation. It exists as two complexes: mTORC1 and mTORC2 [13]. mTOR activation promotes the differentiation of M1‐type macrophages, enhancing the production of inflammatory mediators [14], and it also contributes to the activation of the NOD‐like receptor pyrin domain‐containing 3 (NLRP3) inflammasome, a central driver of intestinal inflammation [15].

NLRP3 is part of a broader family of pattern recognition receptors (PRRs), including NLRP1, NLRP2, NLRP6, NLRP7, NLRP12, and other members such as AIM2 and pyrin [16]. Emerging evidence suggests a regulatory cross talk between AMPK, mTOR, and NLRP3 pathways, where AMPK activation inhibits both mTOR and NLRP3 activity, thus suppressing inflammation [17].

Current pharmacological treatments for UC—including amino salicylates, corticosteroids, immunosuppressants, and biologics—often result in significant adverse effects [18]. Consequently, there is a pressing need to explore safer and more effective therapeutic options. Drug repurposing, or repositioning, is a cost‐effective strategy that identifies new therapeutic uses for existing drugs. This approach has shown promise in the treatment of various diseases, including colon cancer, breast cancer, depression, Parkinson's disease, and UC [19, 20, 21, 22].

Pentoxifylline (PTX), a methylxanthine derivative, possesses notable antioxidant and anti‐inflammatory properties. It reduces blood viscosity by inhibiting platelet and erythrocyte aggregation, lowering plasma fibrinogen levels, enhancing erythrocyte flexibility, and suppressing neutrophil activation [23]. Previous studies have demonstrated the protective effects of PTX in experimental models of colitis, where it mitigated disease severity [24, 25]. However, to date, no studies have investigated the impact of PTX on IL‐6/STAT3, AMPK/mTOR/NLRP3, and Nrf2/HO‐1 pathways in UC.

Mesalamine remains the first‐line therapy for mild to moderate UC and is considered a cornerstone of long‐term disease management. Although generally well tolerated, ongoing research aims to identify adjunct agents that can improve therapeutic outcomes. Preclinical and clinical data suggest that combination therapy with PTX and mesalamine yields superior anti‐inflammatory, antioxidant, and immunomodulatory effects compared to mesalamine alone, without notable side effects [24, 26].

Based on this evidence, the current study aimed to evaluate the therapeutic effects of PTX in an AA‐induced model of UC in rats. Specifically, we investigated the role of PTX in modulating SPHK1/S1P, IL‐6/STAT3, AMPK/mTOR/NLRP3, and ZO‐1 signaling pathways. Additionally, we examined the potential synergistic effects of PTX when administered in combination with mesalamine to enhance therapeutic efficacy in UC.

## 2 Materials and Methods

The College of Veterinary Medicine at Cairo University (Cairo, Egypt) provided 35 male Wistar albino rats, each weighing between 170 g and 210 g, sourced from their animal facility. The rats were housed in standard rat cages under controlled conditions, with a 12‐h light/dark cycle, a temperature of 22°C ± 3°C, 30%–70% relative humidity, and free access to purified water and pelleted food. The rats were allowed 1 week to acclimate to the environment before being used in the experiments. All experimental protocols were approved by the Institutional Review Board (Faculty of Pharmacy, Tanta University, Egypt; Approval number PT‐7‐23‐56) and adhered to the ethical guidelines for the use and care of laboratory animals.

### 2.1 Drugs and Chemicals

For this study, the following products were purchased from the specified vendors: AA was obtained from Chema‐Jet Chemical Company (Alexandria, Egypt) and prepared as a 3% (v/v) solution in normal saline for intracolonic instillation. Mesalamine was sourced from the Egyptian pharmaceutical company EL‐Pharonia, and PTX was acquired from Egyptian Sanofi Pharmaceutical Company. All other chemicals and reagents were purchased from Sigma‐Aldrich. All solvents used in the experiments were of analytical grade.

### 2.2 Induction of Colitis

As previously described [27], colitis was induced in rats by the following method: The rats were allowed free access to water but fasted overnight prior to the induction. Thiopental sodium (20 mg/kg, intraperitoneal) was administered for mild anesthesia. A 2‐mm polypropylene tube was carefully inserted through the rectum into the colon, approximately 8 cm in distance. Two milliliters of a 3% (v/v) AA solution were gradually instilled into the colon. To minimize the risk of premature leakage, the rats were placed in the inverted Trendelenburg position for 30 s following the instillation.

The rats were then randomly assigned to five experimental groups: normal control; colitis control; mesalamine treated group: rats received mesalamine (100 mg/kg; dissolved in 0.9 saline orally daily for 8 days) [28] (2); PTX‐treated group: rats received PTX (100 mg/kg, orally for 8 days) (3); PTX and mesalamine group: rats received PTX (100 mg/kg, orally for 8 days) and mesalamine (100 mg/kg; dissolved in 0.9 saline orally daily for 8 days). The mesalamine and PTX were administered via oral gavage. The normal control group received normal saline instead of AA. All rats, except those in the normal control group, received either the vehicle or their respective therapy for eight consecutive days, beginning 48 h after UC induction.

### 2.3 Quantitative Evaluation of UC

Animal body weights, incidence of diarrhea, and rectal bleeding were recorded twice a week throughout the trial. The Disease Activity Index (DAI) was calculated by averaging the three primary signs: weight loss, diarrhea, and rectal bleeding [29].

$\mathrm{DAI}=\left[\%\text{body weight loss}+\text{diarrhea score}+\text{rectal bleeding score}\right]/3.$

Diarrhea was identified by the presence of stool or mucus adhering to the anal hair, while rectal bleeding was indicated by visible blood in the diarrhea, with or without mucus. Both diarrhea and rectal bleeding were scored as 0 or 1, depending on their presence or absence.

At the end of the study, body weight, colon weight, and colon length were measured. The rats were fasted overnight before killing. Twenty‐four hours after the final dose of PTX, mesalamine, or both, the rats were anesthetized with diethyl ether. Blood was collected via cardiac puncture using a heparinized syringe. Following blood collection, the rats were killed by cervical dislocation under light ether anesthesia.

The colons were carefully excised, excess water was removed by blotting with filter paper, and the colons were rinsed with ice‐cold phosphate‐buffered saline. The colons were then gently stretched, and the length from the distal rectum to the colocecal junction was measured [28]. The distal 8 cm of the colon were divided into two halves: one half was stored at −80°C for biochemical analysis and quantitative real‐time PCR, and the other half was preserved for histological examination.

### 2.4 Macroscopic Examination and Grading of UC

Following postmortem laparotomy and killing, a 6‐cm segment of the colon, extending approximately 2 cm above the anal edge, was excised. This section was then slit lengthwise, and macroscopic abnormalities in the colonic mucosa were evaluated and graded using a scoring system ranging from 0 to 4 [27]. The grading scale was as follows:

- 0: No macroscopic changes
- 1: Mucosal erythema alone
- 2: Mild mucosal edema, slight bleeding, or small erosions
- 3: Moderate edema, bleeding ulcers, or erosions
- 4: Severe ulcerations/erosions, edema, and tissue necrosis

### 2.5 Preparation of Colon Homogenate

Approximately 2 cm of colon tissue was used to prepare a colon homogenate (10% w/v) in an ice‐cold KCl (1.15%, pH 7.4). The homogenate was subjected to centrifugation at 4000 rpm for 30 min at 4°C. After centrifugation, the supernatant was carefully decanted and used for the measurement of colon IL‐6, STAT3, HO‐1, Nrf2, mTOR, S1P, and SPHK.

### 2.6 Assay of SPHK1 Activity

A 50 mg sample of colon tissue was homogenized using a Teflon homogenizer, followed by short sonication (3–15 s) in lysis buffer containing 50 mM HEPES‐NaOH (pH 7.5), 150 mM NaCl, 1 mM DTT, 1 mM EDTA, 1 mM PMSF, and 10% glycerol. The resulting homogenate was then centrifuged at 1000 g for 3 min at 4°C to remove debris. The supernatant was transferred and stored at −80°C until further analysis. To measure SPHK1 activity, 10 μL of the homogenate was processed using rat SPHK1 activity ELISA kits (Echelon Biosciences Inc., K‐3500), following the manufacturer's instructions. SPHK1 protein activity was expressed as pmol/min/mg.

### 2.7 Quantification of Colon Contents of Nrf2 and Colon HO‐1 Activity

ELISA assay kits from Cloud‐Clone Co. (Houston, USA) were used to measure the levels of Nrf2 and the activity of HO‐1 in colon tissue. The manufacturer's instructions were strictly followed for both assays.

### 2.8 Quantification of Colon Contents of STAT3, AMPK, S1P, mTOR, and IL‐6

The levels of IL‐6, mTOR, and STAT‐3 in colon tissue homogenates were assessed using an ELISA kit from Sun Red Biotechnology Co. Ltd. (Shanghai, China) following the manufacturer's instructions. A Labnics Equipment (Fremont, California) ELISA plate reader was used to detect color intensity at 450 nm. The levels of IL‐6, mTOR, and STAT‐3 were quantified and expressed in pg/mg. Additionally, SIP and AMPK levels were measured according to the manufacturer's instructions using an ELISA kit from MyBioSource Co. Ltd. The color intensity for SIP and AMPK was measured at 450 nm and expressed in pg/mg and ng/mg, respectively.

### 2.9 Quantitative Real‐Time Polymerase Chain Reaction (qRT‐PCR) for NLRP3 and ZO‐1

The mRNA expression of NLRP3 and ZO‐1 was quantified using reverse transcription polymerase chain reaction (RT‐PCR), with Glyceraldehyde 3‐phosphate dehydrogenase (GAPDH) as the internal control for standardization. Total RNA was extracted from colonic tissues using Trizol reagent (Sigma, St. Louis, MO, USA) and converted into cDNA. The resulting cDNA was then used as a template for PCR amplification. The primers for the target genes are listed in Table 1. The 30‐cycle amplification process consisted of three steps: denaturation at 95°C for 30 s, annealing at 55°C for 45 s, and extension at 72°C for 1 min. The initial denaturation was performed for 3 min at 95°C, and the final extension was done at 72°C for 7 min.

**TABLE 1:** Primers sequence for selected gene.

Gene	Forward primer (/5————/3)	Reverse primer (/5————/3)
NLRP3	GAGCTGGACCTCAGTGACAA	ACCAATGCGAGATCCTGACA
ZO‐1	GAGGCTTCAGAACGAGGCTA	TGCTTCGGCTCAGATGACTT
GAPDH	TCAAGAAGGTGGTGAAGCAG	AGGTGGAAGAATGGGAGTTG

### 2.10 Histopathological Examination and Immunostaining of Caspase‐3 in Colon Sections

Colon tissue segments were fixed in 10% buffered formalin and embedded in paraffin. After sectioning the tissue into 3 μm slices, the sections were stained with hematoxylin and eosin (H&E) for histological evaluation. Immunohistochemical staining for active Caspase‐3 was performed on 4 μm sections following antigen retrieval using the Heat Induced Epitope Retrieval (HIER) method with Cell Marque Trilogy. The sections were incubated with primary antibodies against active Caspase‐3 (rabbit polyclonal, 1:1000) for 60 min, followed by incubation with Ultra Vision One HRP Polymer for 15 min. The antigen was visualized using a DAB substrate solution, and sections were counterstained with hematoxylin.

The slides were imaged using an MEIJI MX5200L microscope with a digital eyepiece (MVV5000CL) and analyzed using Fiji ImageJ software (version 1.51r). The Color Deconvolution 2 plugin was employed to separate the histological dyes and quantify the staining area, with the percentage of positive staining calculated from five randomly selected fields (200 × 200 μm). Data were exported to Excel for further statistical analysis.

### 2.11 Statistical Analysis

All statistical analyses were conducted using Prism version 9 (GraphPad Software Inc., San Diego, California, USA). The normality of continuous variables was assessed using the Shapiro–Wilk test. Data are presented as means ± standard deviation (SD). For comparison of parametric data, one‐way analysis of variance (ANOVA) was used, followed by multiple comparisons using the Tukey–Kramer test. For nonparametric data, the Kruskal–Wallis test was applied, followed by Dunn's multiple comparison test. Body weight differences within groups were assessed using a paired Student's t‐test. Statistical significance was set at *p* < 0.05, with all *p*‐values being two‐tailed.

## 3 Results

### 3.1 Effect of Mesalamine, PTX, and Their Combination on AA‐Induced Changes in Colon Weight, Length, and Body Weight

In the colitis group, colon weight was significantly increased by 1.28‐fold compared to the control group (*F* = 63.11, *p* < 0.0001). Treatment with mesalamine alone resulted in a significant reduction in colon weight by 14.87% compared to the colitis group (*F* = 63.11, *p* = 0.005). Similarly, PTX treatment significantly decreased colon weight by 13.99% compared to the colitis group (*F* = 63.11, *p* = 0.002, *n* = 7). The co‐administration of mesalamine and PTX resulted in a significant decrease in colon weight by 22.6% compared to the colitis group (*F* = 63.11, *p* < 0.0001).

Interestingly, the combination treatment of mesalamine and PTX also showed a significant reduction in colon weight compared to mesalamine alone by 9.08% (*F* = 63.11, *p* = 0.0005).

When comparing the treatments to the normal control group, colon weight was significantly increased in both the mesalamine (*F* = 63.11, *p* = 0.0007) and PTX (*F* = 63.11, *p* = 0.0001) groups compared to the normal group. However, there was no significant difference in colon weight between the combination treatment and the normal control group (*p* > 0.9999) indicating near‐complete normalization of colon weight with the combined therapy.

In summary, the weight of the colon increased in the following order: colitis group > PTX group > mesalamine group > combination group ≈ normal control group.

**FIGURE 1:**
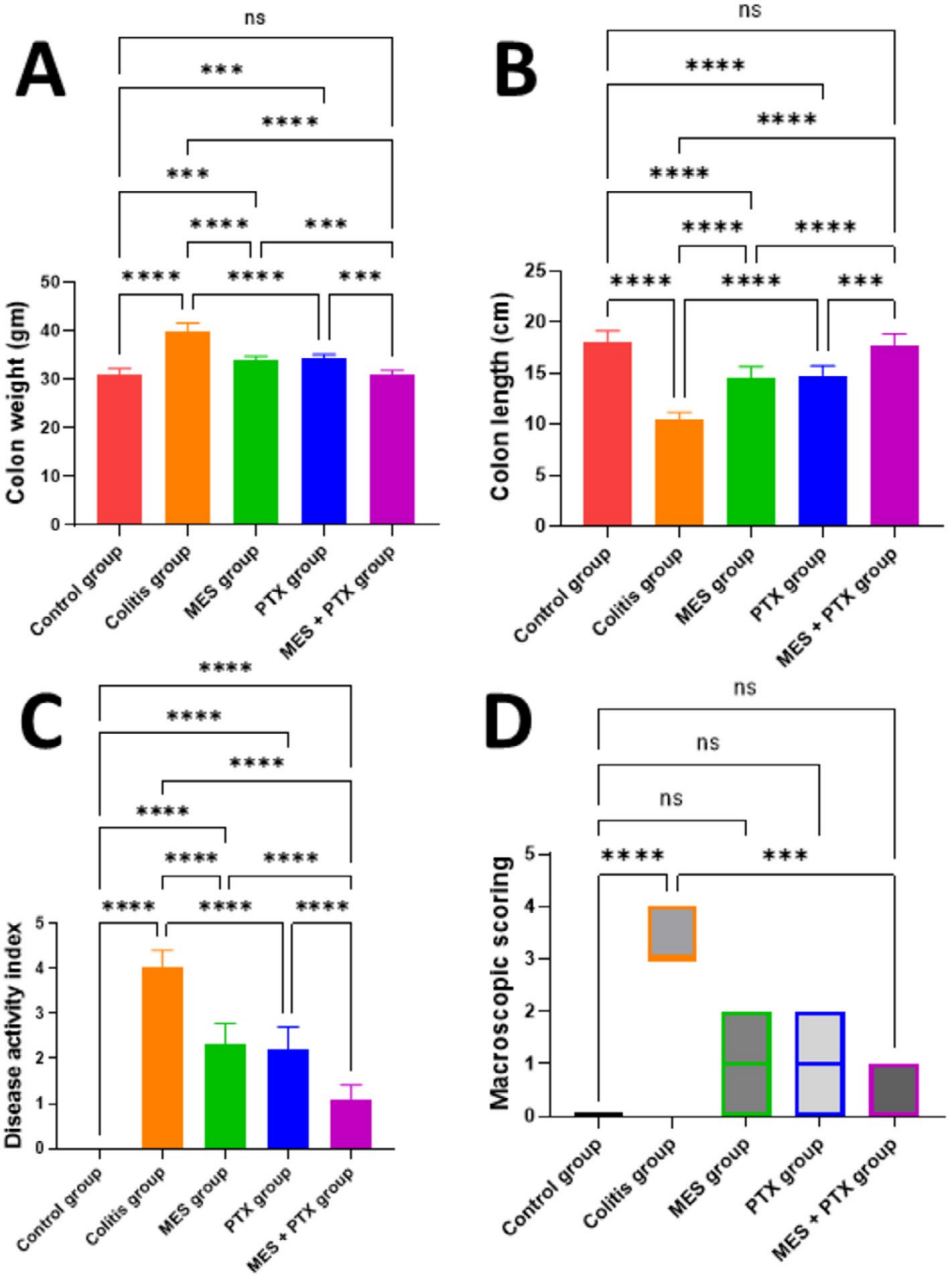
Effect of MES, PTX, and their combination on acetic acid‐induced change in colon weight (A), colon length (B), disease activity index (C), and macroscopic scoring (D). MES, mesalamine; PTX, pentoxifylline. Data are presented as mean and standard deviation, (ns) nonsignificant, significance at *p* < 0.05. (***) indicates *p* < 0.001, (****) indicates *p* < 0.0001.

Both colon length and overall body weight were significantly decreased in the colitis group by 41.57% (*F* = 59.82, *p* < 0.0001) and 23.69% (*F* = 63.34, *p* < 0.0001), respectively, compared to the normal control group. Treatment with mesalamine significantly increased colon length and body weight by 1.38‐fold (*F* = 59.82, *p* = 0.007) and 1.22‐fold (*F* = 63.34, *p* = 0.02), respectively, compared to the colitis group. Similarly, PTX treatment significantly increased colon length and body weight by 1.40‐fold (*F* = 59.82, *p* = 0.006) and 1.20‐fold (*F* = 63.34, *p* = 0.008), respectively, compared to the diseased group (Figure 1A,B).

The combination treatment of mesalamine and PTX showed significant increases in both colon length and body weight, with increases of 1.67‐fold (*F* = 59.82, *p* < 0.0001) and 1.31‐fold (*F* = 63.34, *p* < 0.0001), respectively, compared to the colitis group. Compared to the mesalamine‐only group, the combination treatment also resulted in significant increases of 1.20‐fold (*F* = 59.82, *p* < 0.0001) in colon length and 1.07‐fold (*F* = 63.34, *p* = 0.0043) in body weight.

The colon length was significantly increased in the normal control group compared to both the PTX (*F* = 59.82, *p* = 0.0001) and mesalamine (*F* = 59.82, *p* = 0.0001) groups. However, no significant changes were observed when compared to the combination treatment group (*p* = 0.970). In contrast, no significant changes were observed in total body weight between PTX, mesalamine, or their combination when compared to the normal control group (Table 2).

**TABLE 2:** Effect of studied drugs on body weight of animals.

Body weight (g)	Before treatment	After treatment	*p*
Control group	188.3 ± 8.381	194.1 ± 3.530	0.0001
Colitis group	191.9 ± 9.651	177 ± 7.916	0.0001
MES group	189.7 ± 9.232	195.3 ± 6.130	0.0001
PTX group	187.4 ± 9.413	193 ± 4.967	0.0002
MES + PTX group	188 ± 7.810	192.7 ± 3.964	0.0002

*Note:* Data are presented as mean and SD. Significance at *p* < 0.05 using paired *t*‐test.

Abbreviations: MES, mesalamine; PTX, pentoxifylline.

### 3.2 Effect of Mesalamine, PTX, and Their Combination on AA‐Induced Change in Disease Activity Index and Macroscopic Scoring

The colitis group revealed a significant increase in both the Disease Activity Index (DAI) and macroscopic score, with increases of 4.05 folds (*F* = 114.5, *p* < 0.0001) and 3.7 folds (*F* = 114.5, *p* = 0.0001), respectively, compared to the control group. Mesalamine significantly decreased both DAI (44.98%, *F* = 114.5, *p* < 0.0001) and the macroscopic score (96.15%, *F* = 114.5, *p* = 0.0009) when compared to the colitis group. Similarly, the PTX group showed a significant decrease in both parameters, with reductions of 45.69% (*F* = 114.5, *p* < 0.0001) in DAI and 96.14% (*F* = 114.5, *p* = 0.0009) in the macroscopic score compared to the colitis group. The combination therapy significantly reduced DAI by 72.88% (*F* = 114.5, *p* < 0.0001) and the macroscopic score by 96.17% (*F* = 114.5, *p* = 0.0009) in comparison with the diseased group. When comparing the combination treatment to the mesalamine‐only group, there was also a significant reduction in both parameters by 52.58% (*F* = 114.5, *p* < 0.0001) in DAI and 50.06% (*F* = 114.5, *p* < 0.0001) in macroscopic score (Figure 1C,D).

When comparing the normal control group to all treatment groups, there was a significant decrease in DAI (*F* = 114.5, *p* < 0.0001). However, no significant changes were observed in macroscopic scores between the normal control group and PTX (*p* = 0.571), mesalamine (*p* = 0.152), and combination treatment groups (*p* = 0.999).

### 3.3 Effect of Mesalamine, PTX, and Their Combination on AA‐Induced Change in Colon mTOR Content and Colon Gene Expression of NLRP3

The colitis group showed a significant increase in both colon mTOR content and NLRP3 gene expression, with 3.61‐fold (*F* = 528.4, *p* < 0.0001) and 3.68‐fold (*F* = 66.14, *p* < 0.0001) increases, respectively, compared to the normal control group. Treatment with mesalamine significantly reduced mTOR levels by 49.75% (*F* = 528.4, *p* = 0.004) and downregulated NLRP3 expression by 40.32% (*F* = 66.14, *p* = 0.008) compared to the colitis group. Similarly, PTX treatment resulted in significant reductions of 50.08% in mTOR (*F* = 528.4, *p* = 0.02) and 53.93% in NLRP3 expression (*F* = 66.14, *p* = 0.006) versus the colitis group. Notably, the combination therapy led to a more pronounced decrease in both mTOR (67.02%, [*F* = 528.4, *p* < 0.0001]) and NLRP3 (71.23%, [*F* = 66.14, *p* < 0.0001]) levels compared to untreated rats.

**FIGURE 2:**
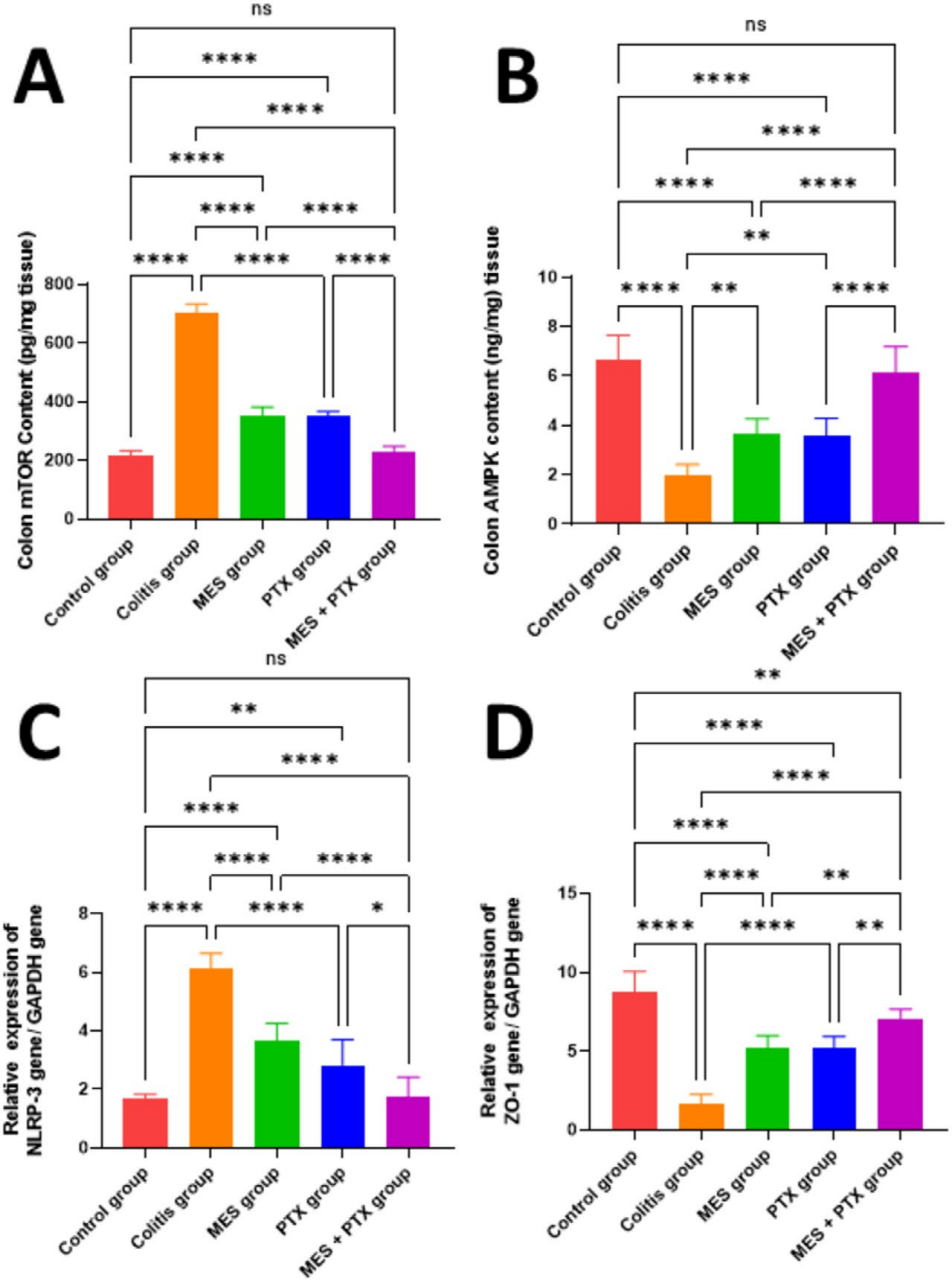
Effect of MES, PTX, and their combination on acetic acid‐induced change in colon mTOR content (A), colon AMPK content (B), NLRP3 gene expression (C), and ZO‐1 (D) gene expression. MES, mesalamine; PTX, pentoxifylline. Data are presented as mean and standard deviation, (ns) nonsignificant, significance at *p* < 0.05. (*) indicates *p* < 0.05, (**) indicates *p* < 0.01, (***) indicates *p* < 0.001, (****) indicates *p* < 0.0001.

Furthermore, the combination treatment demonstrated superior efficacy over mesalamine monotherapy, with significant reductions of 34.36% in mTOR (*F* = 528.4, *p* = 0.002) and 51.79% in NLRP3 (*F* = 66.14, *p* = 0.03) (Figure 2A,C).

Compared to the normal control group, both the PTX and mesalamine groups showed significantly elevated levels of mTOR (*F* = 528.4; *p* = 0.0001 for both) and NLRP3 (*F* = 66.14; *p* = 0.008 and 0.0001, respectively). In contrast, no significant differences were observed between the normal control and combination treatment groups (*p* = 0.747 for mTOR and *p* = 0.997 for NLRP3), suggesting that the combined therapy nearly normalized these molecular markers.

### 3.4 Effect of Mesalamine, PTX, and Their Combination on AA‐Induced Change in Colon ZO‐1 Gene Expression and AMPK Colon Content

The colitis group demonstrated a significant reduction in colon ZO‐1 gene expression and AMPK content by 81.15% (*F* = 65.2, *p* < 0.0001) and 69.89% (*F* = 78.34, *p* < 0.0001), respectively, compared to the normal control group. Treatment with mesalamine significantly upregulated ZO‐1 expression by 3.13‐fold (*F* = 65.2, *p* < 0.0001) and increased AMPK content by 2.32‐fold (*F* = 78.34, *p* < 0.0001) relative to the colitis group. Similarly, PTX treatment resulted in a 3.16‐fold increase in ZO‐1 (*F* = 65.2, *p* < 0.0001) and a 2.36‐fold rise in AMPK levels (*F* = 78.34, *p* < 0.0001) compared to the diseased group.

Notably, the combination therapy produced the most significant improvements, with ZO‐1 gene expression increasing by 4.29‐fold (*F* = 65.2, *p* < 0.0001) and AMPK content by 3.36‐fold (*F* = 78.34, *p* < 0.0001) compared to the colitis group. Moreover, the combination treatment significantly outperformed mesalamine alone, showing a 1.37‐fold higher ZO‐1 expression (*F* = 65.2, *p* = 0.0022) and a 1.45‐fold increase in AMPK levels (*F* = 78.34, *p* < 0.0001), as shown in Figure 2B,D.

When compared to the normal control group, both the mesalamine and PTX groups continued to exhibit significantly reduced levels of ZO‐1 (*F* = 65.2; *p* = 0.0007 and 0.009, respectively) and AMPK (*F* = 78.34; *p* < 0.0001 for both). However, the combination therapy group showed no significant difference in AMPK levels relative to the normal controls (*p* = 0.776), indicating near‐complete normalization. However, ZO‐1 expression remained significantly lower in the combination group compared to the normal group (*F* = 65.2; *p* = 0.009).

### 3.5 Effect of Mesalamine, PTX, and Their Combination on AA‐Induced Change in Colon SPHK and S1P Content

**FIGURE 3:**
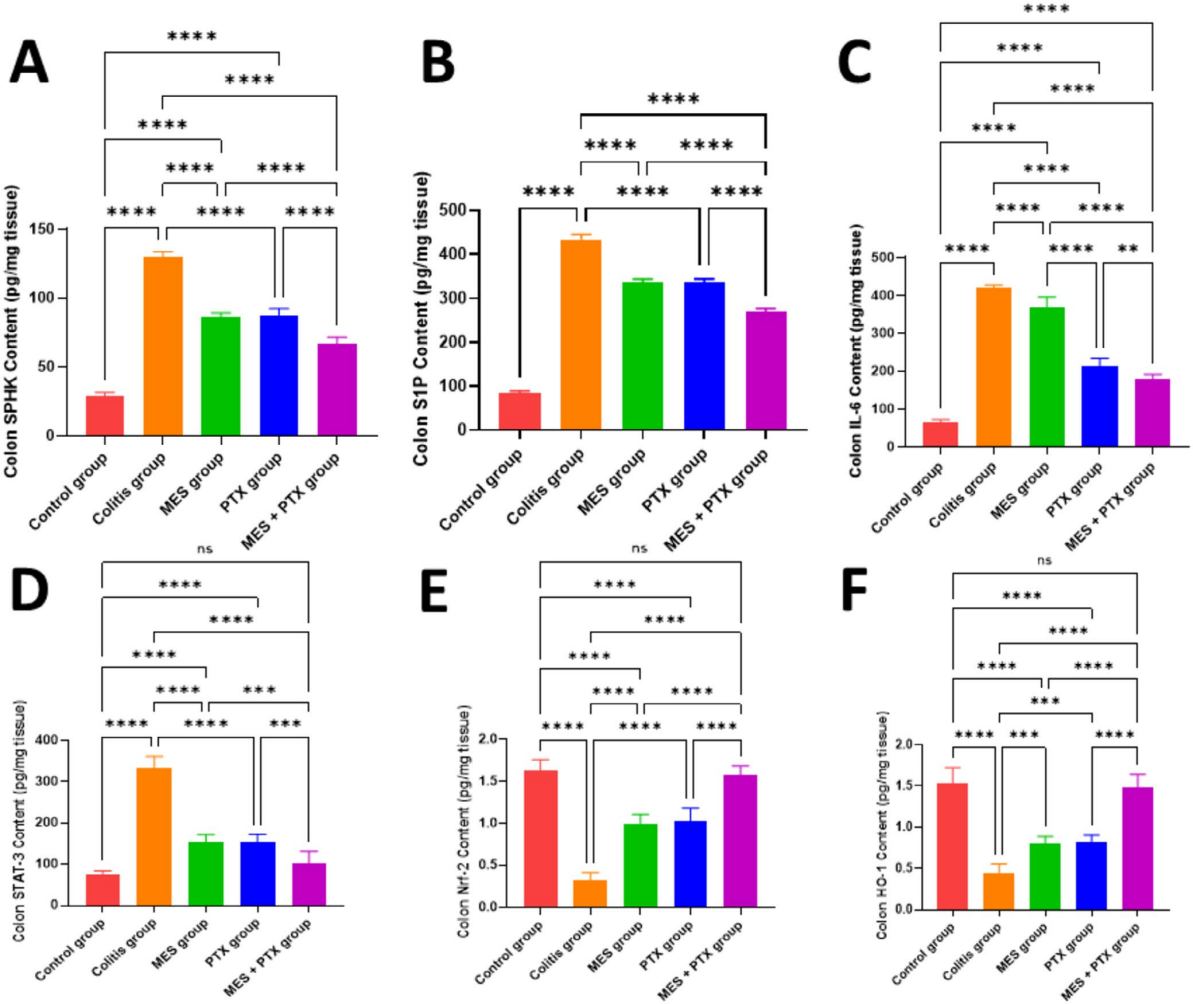
Effect of MES, PTX, and their combination on acetic acid‐induced change in SPHK (A), S1P (B), IL‐6 (C), STAT3 (D), Nrf‐2 (E), and HO‐1 (F). MES: mesalamine; PTX: pentoxifylline. Data are presented as mean and standard deviation, (ns) nonsignificant, significance at *p* < 0.05. (*) indicates *p* < 0.05, (**) indicates *p* < 0.01, (***) indicates *p* < 0.001, and (****) indicates *p* < 0.0001.

As shown in Figure 3A,B, the colitis group exhibited a significant elevation in colon SPHK and S1P content by 4.46‐fold (*F* = 582.9, *p* < 0.0001) and 5.11‐fold (*F* = 1755, *p* < 0.0001), respectively, compared to the normal control group. Treatment with mesalamine significantly reduced SPHK and S1P levels by 34.04% (*F* = 582.9, *p* = 0.0004) and 22.64% (*F* = 1755, *p* = 0.003), respectively, relative to the colitis group. Similarly, PTX treatment led to a significant reduction in SPHK by 32.6% (*F* = 582.9, *p* = 0.0002) and S1P by 22.48% (*F* = 1755, *p* = 0.004), compared to the diseased group.

Co‐administration of mesalamine and PTX produced a more pronounced effect, significantly reducing SPHK and S1P content by 48.89% (*F* = 582.9, *p* < 0.0001) and 38.01% (*F* = 1755, *p* < 0.0001), respectively, in comparison with the colitis group. Moreover, when compared to the mesalamine group alone, the combination therapy further decreased SPHK by 22.52% (*F* = 582.9, *p* < 0.0001) and S1P by 19.86% (*F* = 1755, *p* < 0.0001).

Interestingly, all treatment groups (mesalamine, PTX, and combination) continued to exhibit significantly elevated SPHK and S1P levels compared to the normal control group (all *p* < 0.0001), indicating that none of the treatments fully normalized these markers.

### 3.6 Effect of Mesalamine, PTX, and Their Combination on AA‐Induced Change in Colon IL‐6 and STAT‐3 Content

As illustrated in Figure 3C,D, the colitis group demonstrated a significant increase in colon IL‐6 and STAT3 levels by 6.77‐fold (*F* = 505.4, *p* < 0.0001) and 4.38‐fold (*F* = 153.3, *p* < 0.0001), respectively, compared to the normal control group. Treatment with mesalamine resulted in a modest but significant reduction in IL‐6 and STAT3 content by 12.71% (*F* = 505.4, *p* = 0.006) and 53.57% (*F* = 153.3, *p* = 0.004), respectively, relative to the colitis group. Similarly, PTX treatment led to significant decreases in IL‐6 and STAT3 by 49.66% (*F* = 505.4, *p* < 0.0001) and 53.51% (*F* = 153.3, *p* = 0.001), respectively.

The combined administration of mesalamine and PTX produced the most pronounced effect, significantly reducing IL‐6 and STAT3 levels by 57.93% (*F* = 505.4, *p* < 0.0001) and 68.91% (*F* = 153.3, *p* < 0.0001), respectively, in comparison to the colitis group. Furthermore, when compared to the mesalamine group alone, the combination therapy further reduced IL‐6 by 51.8% (*F* = 505.4, *p* < 0.0001) and STAT3 by 33.03% (*F* = 153.3, *p* = 0.0009).

Despite these improvements, both the mesalamine and PTX groups continued to show significantly elevated levels of IL‐6 (*F* = 505.4; *p* < 0.0001) and STAT3 (*F* = 153.3; *p* < 0.0001 for both) compared to the normal control group. Notably, the combination therapy group exhibited no significant difference in STAT3 levels relative to the normal controls (*p* = 0.138), indicating near‐complete normalization. However, IL‐6 levels remained significantly elevated in the combination group (*F* = 505.4; *p* < 0.0001), suggesting only partial resolution of inflammation with the combined treatment.

### 3.7 Effect of Mesalamine, PTX, and Their Combination on AA‐Induced Change in Colon Nrf‐2 and HO1 Content

As shown in Figure 3E,F, the colitis group exhibited a marked reduction in colon Nrf2 and HO‐1 levels by 79.96% (*F* = 128.2, *p* < 0.0001) and 73.11% (*F* = 141.7, *p* < 0.0001), respectively, compared to the normal control group. Treatment with mesalamine significantly increased Nrf2 and HO‐1 levels by 3.01‐fold (*F* = 128.2, *p* = 0.0003) and 1.79‐fold (*F* = 141.7, *p* = 0.02), respectively, relative to the colitis group. Similarly, PTX significantly elevated Nrf2 and HO‐1 contents by 3.13‐fold (*F* = 128.2, *p* = 0.0007) and 1.81‐fold (*F* = 141.7, *p* = 0.01), respectively, compared to the diseased group.

Combined treatment with mesalamine and PTX resulted in a 2.8‐fold increase in Nrf2 (*F* = 128.2, *p* < 0.0001) and a 3.28‐fold increase in HO‐1 (*F* = 141.7, *p* < 0.0001), significantly higher than those observed in the colitis group. Furthermore, this combination therapy produced significantly greater levels than mesalamine monotherapy, with Nrf2 and HO‐1 increased by 1.59‐fold (*F* = 128.2, *p* = 0.002) and 1.83‐fold (*F* = 141.7, *p* = 0.0009), respectively.

Although both mesalamine and PTX monotherapies resulted in improvements, the levels of Nrf2 (*F* = 128.2; *p* < 0.0001) and HO‐1 (*F* = 128.2; *p* < 0.0001) remained significantly lower than those in the normal control group. In contrast, the combination therapy group showed no significant differences in Nrf2 (*p* = 0.883) or HO‐1 (*p* = 0.253) levels compared to the normal controls, indicating complete normalization and suggesting optimal therapeutic efficacy.

### 3.8 Effect of Mesalamine, PTX, and Their Combination on AA‐Induced Change in Colon Caspase‐3 Expression

**FIGURE 4:**
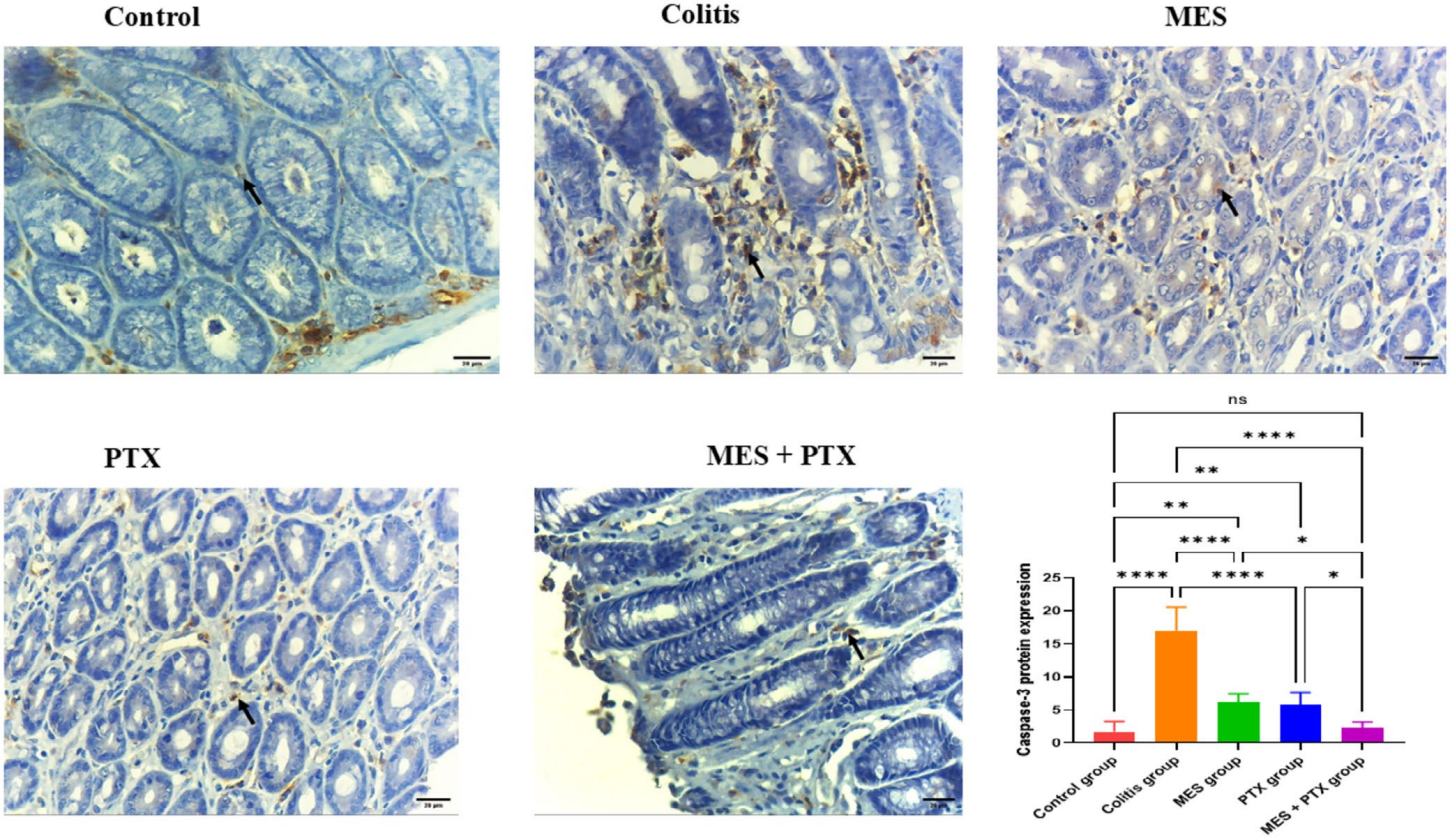
Effect of MES, PTX, and their combination on acetic acid‐induced change in caspase 3 expression. MES: mesalamine; PTX: pentoxifylline. Data were presented as mean and standard deviation, (ns) non‐significant, significance at *p* < 0.05. (*) indicates *p* < 0.05, (**) indicates *p* < 0.01, (***) indicates *p* < 0.001, and (****) indicates *p* < 0.0001. Caspase 3 immunohistochemical staining micrographs of colon sections of (A) Normal control colon, showing few cytoplasmic positive cells in the lamina propria. (B) Ulcerative colitis, showing several cytoplasmic positive cells with high intensity in the lamina propria. (C) Mesalamine, showing decrease in the number of the positive cells. (D) PTX group, showing decrease number of positive cells, (E) Combination treatment, showing few to nonpositive cells in the lamina propria.

As illustrated in Figure 4, the colitis group demonstrated a significant increase in colon caspase‐3 expression, showing a 9.95‐fold elevation compared to the normal control group (*F* = 56.53, *p* < 0.0001). Treatment with mesalamine significantly downregulated caspase‐3 expression by 63.83% (*F* = 56.53, *p* < 0.0001), while PTX treatment led to a comparable reduction of 59.5% (*F* = 56.53, *p* < 0.0001) relative to the diseased group.

Coadministration of mesalamine and PTX produced a more pronounced effect, significantly reducing caspase‐3 expression by 89.5% compared to the untreated colitis group (*F* = 44.02, *p* < 0.0001), and by 71.02% when compared to the mesalamine‐only group (*F* = 56.53, *p* = 0.0067).

Interestingly, both the mesalamine and PTX monotherapy groups continued to exhibit significantly higher caspase‐3 expression compared to the normal control group (*F* = 56.53; *p* = 0.003 and 0.007, respectively). In contrast, the combination therapy group showed no significant difference in caspase‐3 expression relative to the normal controls (*p* = 0.981), suggesting near‐complete normalization of apoptotic activity.

### 3.9 Effect of Mesalamine, PTX, and Their Combination on AA‐Induced Change in Histopathological Features of the Colon

Histological analysis of colonic tissues from the normal control group revealed intact mucosal architecture, with well‐organized crypts lined by goblet cells and surrounded by a normal lamina propria. In contrast, colon sections from the colitis group exhibited marked architectural distortion, including irregular crypt formation, intense lymphoplasmacytic infiltration, and prominent fibrosis within the lamina propria.

Treatment with mesalamine led to partial restoration of colonic architecture, characterized by improved crypt structure and minimal inflammatory cell infiltration and fibrosis. Similarly, the PTX‐treated group showed partial restoration of the mucosal architecture, although some irregular crypts and fibrosis in the lamina propria were still observed.

**FIGURE 5:**
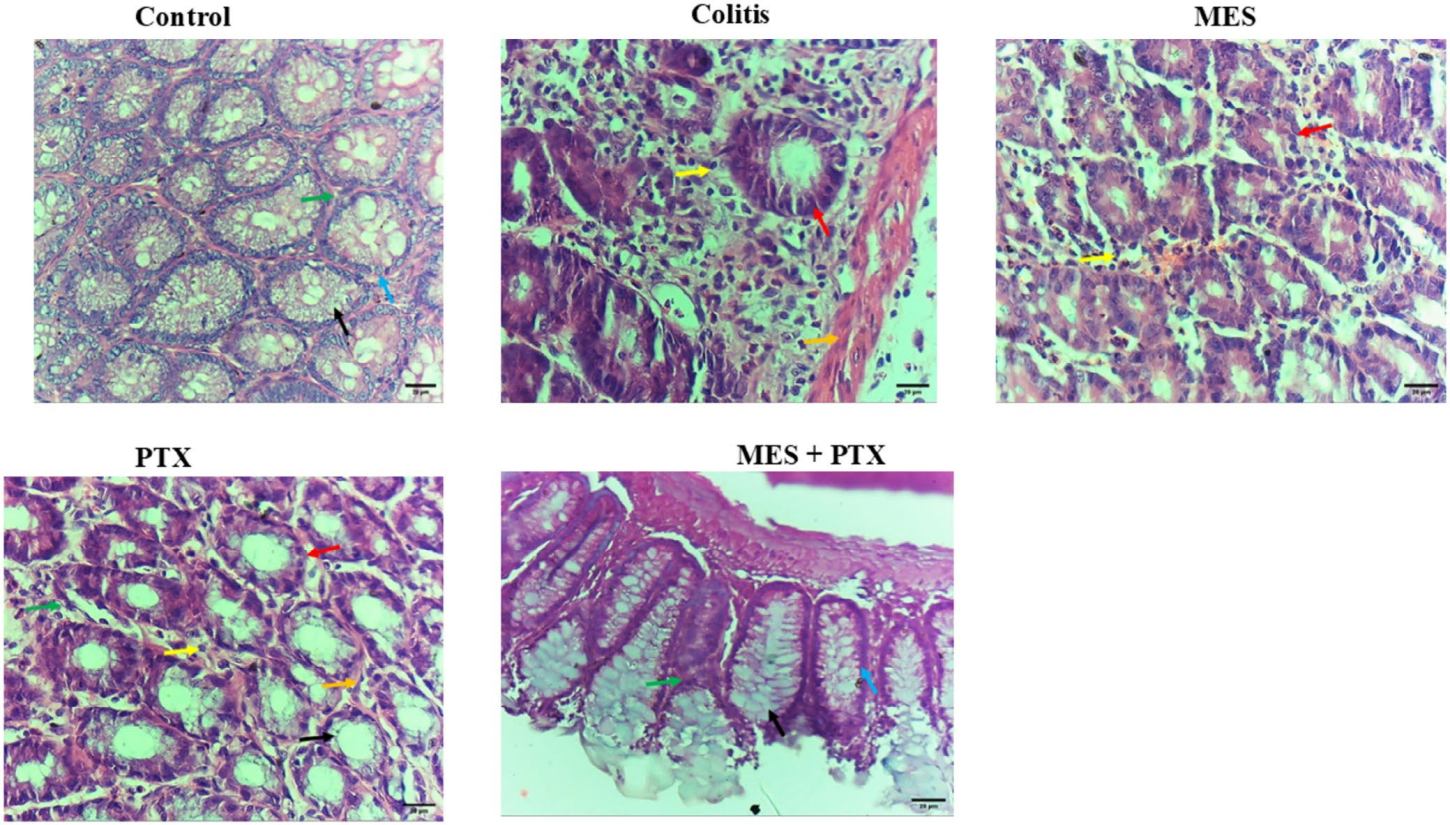
H&E staining micrographs of colon sections of (A) Normal control colon, showing colon crypts lined by goblet cells and surrounded by the lamina propria. (B) Ulcerative colitis, showing distortion of the normal architecture with irregular crypts, intense lymphoplasmacytic infiltration, and intense fibrosis in the lamina propria. (C) Mesalamine group, showing partial restoration of the normal architecture and minimal lymphoplasmacytic infiltration and fibrosis in the lamina propria. (D) PTX group, showing partial restoration of the normal architecture with irregular shaped crypts, and fibrosis in the lamina propria. (E) Combination treatment group, showing complete restoration of the normal architecture with normal shaped crypts, and minimal fibrosis in the lamina propria. Black arrow: goblet cell, blue arrow: crypt, green arrow: lamina propria, yellow arrow: lymphoplasmacytic infiltration, red arrow: irregular crypts, orange arrow: fibrosis. 400× magnification.

Notably, colonic sections from the combination therapy group demonstrated near‐complete restoration of normal histological features, including well‐formed crypts and markedly reduced fibrosis, with minimal inflammatory infiltrates in the lamina propria (Figure 5).

## 4 Discussion

The present study demonstrated that UC induced a significant degree of macroscopic injury, functional impairment, and biochemical alterations, culminating in compromised colonic function and clinical indices. These findings are consistent with previous reports [27, 30, 31]. The AA‐induced model of UC in experimental animals remains a widely accepted and standardized approach for mimicking the pathophysiological features of human UC, including inflammation, oxidative stress, and disruption of mucosal integrity [32]. This model is characterized by elevated expression of inflammatory cytokines, enhanced lipid peroxidation, and impaired antioxidant defense mechanisms within the mucosa [27].

In this study, treatment with either mesalamine, PTX, or their combination significantly ameliorated DAI, histopathological alterations, and improved clinical outcomes such as body weight gain, reduced colon weight, and increased colon length. These results are in accordance with earlier studies [25, 33]. Notably, PTX mitigated mucosal injury by modulating tissue biomarkers associated with inflammation, oxidative stress, and fibrosis, supporting its potential as an adjunctive therapy in experimental models of colitis [25].

Several experimental studies have investigated the therapeutic potential of PTX in models of colitis; however, the present study is the first to elucidate its modulatory effects on the SPHK1/S1P, IL‐6/STAT3, AMPK/mTOR/NLRP3, and ZO‐1 signaling pathways in experimental colitis. In the current model, both PTX and mesalamine significantly reduced colonic levels of S1P and SPHK1 compared to the untreated colitis group. Consistent with earlier findings [28, 34], the colitis group exhibited markedly elevated levels of S1P and SPHK1 compared to healthy controls. Activation of the SPHK1 pathway is known to potentiate inflammatory and immune responses within the intestinal mucosa [35]. Supporting its pathogenic role in UC, Abdin et al. reported a strong positive correlation between DAI and SPHK1 expression [36].

The mechanisms through which PTX modulates serum S1P levels may be multifaceted. TNF‐α is known to activate SPHK, a lipid kinase responsible for the phosphorylation of sphingosine to generate S1P, a key bioactive lipid involved in mucosal inflammation [37]. PTX is thought to exert its effects by activating protein kinase A (PKA) and increasing intracellular cyclic AMP (cAMP), ultimately leading to inhibition of RhoA [38]. The observed reduction in serum S1P levels following PTX administration may therefore reflect an inhibitory effect on SPHK1 activity. This proposed mechanism is in line with previous reports documenting PTX's potent suppression of TNF‐α production [39, 40]. Hence, the findings of this study support the hypothesis that PTX attenuates SPHK1/S1P signaling via inhibition of TNF‐α and activation of the cAMP/PKA axis, contributing to its anti‐inflammatory effects in colitis.

In the present study, colonic levels of IL‐6 and STAT3 were significantly decreased in the PTX‐treated, mesalamine‐treated, and combination‐treated groups compared to the colitis group. These findings are consistent with previous studies that reported the downregulation of the IL‐6/STAT3 signaling pathway as a key mechanism in the mitigation of colitis severity [41, 42]. Han et al. demonstrated that inhibition of IL‐6/STAT3 signaling led to a marked reduction in colitis severity, supporting the role of this pathway in disease pathogenesis [6].

PTX has been shown to suppress both constitutive and IL‐6‐induced STAT3 activation in a dose‐dependent manner, as observed in A375 melanoma cells. This effect is mediated in part through the inhibition of upstream Janus kinases (pJAK1 and pJAK2) [43]. Upon activation, STAT3 undergoes phosphorylation at Tyr705, dimerizes, and translocates to the nucleus, where it binds to specific promoter regions of its target genes [44]. Treatment with PTX was found to inhibit both STAT3 phosphorylation and its binding to DNA, indicating a robust capacity to block STAT3 activation [43]. Additionally, PTX may exert indirect inhibitory effects on STAT3 by downregulating hypoxia‐inducible factor 1‐alpha (HIF‐1α), further supporting its modulatory role in inflammatory signaling [43]. Other studies have also linked the inhibitory effects of PTX on the IL‐6/STAT3 axis to its ability to suppress the nuclear factor kappa B (NF‐κB) pathway [45], a central regulator of proinflammatory gene expression. These collective mechanisms highlight PTX's multifaceted anti‐inflammatory properties and its potential to ameliorate colonic inflammation through targeting key molecular pathways.

The present study demonstrated that treatment with AA led to significant disruption of tight junction proteins, as evidenced by reduced expression of ZO‐1 in comparison with the healthy control, PTX, and mesalamine groups. Both the PTX and mesalamine treatments, as well as the combination therapy, significantly increased the expression of ZO‐1 compared to the colitis group. These findings are consistent with prior studies investigating the impact of PTX and mesalamine on tight junction proteins [28, 46, 47].

The observed effects of PTX on intestinal ZO‐1 expression may be attributed to modifications in intracellular signaling pathways. Several signaling cascades, including calcium, Ras, Rho, cAMP, and PKA, regulate the permeability of the epithelial barrier [48]. PTX is known to inhibit phosphodiesterase (PDE), leading to an increase in intracellular cAMP levels. Furthermore, PTX acts through both PKA‐dependent and independent mechanisms [49, 50]. However, its immunomodulatory effects likely cannot be explained by PDE inhibition alone [51]. PTX may also influence tight junction protein regulation through the mitogen‐activated protein kinase (MAPK) signaling pathway, which plays a critical role in the phosphorylation and dephosphorylation of ZO‐1 [52]. Previous studies have demonstrated that PTX can attenuate inflammation‐related signaling by inhibiting p38 MAPK and extracellular signal‐regulated kinase (ERK) activation in human neutrophils [53].

Proinflammatory cytokines such as TNF‐α and IL‐1β have been shown to promote ZO‐1 degradation, increasing tight junction permeability. Ma et al. reported that TNF‐α induced a downregulation of ZO‐1 and heightened intestinal permeability, which was mediated by NF‐κB activation [54]. In this study, PTX exhibited strong inhibitory effects on TNF‐α release and NF‐κB activation, thereby preventing the breakdown of ZO‐1 [55]. The combination therapy of mesalamine and PTX significantly upregulated ZO‐1 expression, providing enhanced protection to the intestinal mucosal barrier compared to monotherapy. This confirms the beneficial effects of combination therapy in maintaining epithelial integrity, as supported by the findings of the current study.

In the present study, the PTX group exhibited a significant increase in colonic AMPK expression and a significant decrease in colonic expression of NLRP3 and mTOR when compared to the colitis group. These findings are consistent with previous research [56, 57, 58]. The mTOR/NLRP3 inflammasome pathway is a crucial downstream signaling pathway regulated by AMPK. As previously reported, reduced phosphorylation of AMPK can lead to increased mTOR activity [17]. AMPK exerts its effects by phosphorylating key regulators such as tuberous sclerosis protein 2 (TSC2), Raptor, and mTORC1, thereby suppressing their activity. This suppression of TSC2 and mTORC1 prevents excessive activation of mTOR and, in turn, limits the upregulation of the NLRP3 inflammasome. When AMPK activity is reduced, mTOR activity is elevated, which subsequently triggers NLRP3 inflammasome activation, largely through ROS‐induced overexpression of NLRP3 [59].

Gregory et al. demonstrated that PTX indirectly reduces mTOR expression by inhibiting TNF‐α release. Their findings suggested that TNF‐α activates mTOR in astrocytes, and this pathway is effectively blocked by PTX and propentofylline. Moreover, PTX inhibits the activation of JNK and p38 pathways, thereby modulating the signaling cascades involved in mTOR activation [57]. Additionally, PTX has known antifibrotic effects by inhibiting the tumor growth factor‐beta (TGF‐β) signaling cascade, which plays a pivotal role in mTOR activation [60]. It has been reported that TGF‐β can activate the mTOR pathway [61]. PTX also activates AMPK and suppresses the NF‐κB pathway, leading to a downregulation of both mTOR and NLRP3 expression in the colonic mucosa [62, 63]. These findings further support the role of PTX in modulating key inflammatory and fibrotic pathways involved in UC.

Nuclear erythroid 2‐related factor 2 (Nrf2) is a crucial oxidative stress sensor that regulates the expression and coordinated activation of various protective genes, including those encoding antioxidant proteins and detoxification enzymes [64]. According to Kim et al., the protection against inflammatory‐induced tissue damage relies on the coordinated expression of antioxidative and cytoprotective genes through the activation of the Nrf2‐ARE system [65]. Activation of the Nrf2/ARE signaling pathway has been shown to reduce inflammation, a key factor in many diseases, including UC. In the current study, treatment with PTX and mesalamine significantly restored colon Nrf2 expression. Interestingly, when administered therapeutically, both PTX and mesalamine not only normalized but also enhanced colon Nrf2 levels, which helped mitigate the effects of UC‐induced stress. These findings suggest that PTX‐induced Nrf2 activation plays a significant role in modulating antioxidant defense mechanisms, inflammatory processes, and apoptotic cascades, all of which contribute to the progression and management of UC.

Additionally, Nrf2 induction is believed to enhance the activity of HO‐1. HO‐1 is known for its potent anti‐inflammatory and antioxidative properties, as it prevents the synthesis of proinflammatory mediators. Its elevation is particularly associated with acute inflammatory diseases, including intestinal inflammation [66]. The current study's finding that PTX restored colon HO‐1 activity further underscores the protective role of HO‐1 in maintaining cellular homeostasis and its anti‐inflammatory effects in murine models of UC. These results are consistent with previous research, highlighting the importance of the Nrf2/HO‐1 axis in inflammatory regulation [67, 68].

Abnormal apoptosis has been linked to UC, with increased apoptotic activity contributing to the breakdown of the epithelial barrier, facilitating mucosal invasion by pathogenic bacteria [28]. In the present study, a significant increase in caspase‐3 expression was observed in the UC group, indicating heightened apoptosis. Treatment with PTX, mesalamine, and their combination resulted in a significant reduction in caspase‐3 expression. These findings are consistent with previous studies [69], which have also reported the inhibitory effects of PTX on apoptosis. The antiapoptotic properties of PTX are thought to stem from its ability to reduce oxidative stress and inflammation [69]. In addition, mesalamine treatment in our study similarly decreased caspase‐3 expression in the colonic tissue, aligning with prior research [28]. The antiapoptotic effects of PTX may be attributed to its ability to inhibit the extrinsic apoptotic pathway driven by TNF‐α and the JNK signaling pathway [69]. Additionally, PTX's antioxidant potential plays a key role in mitigating ROS‐induced intrinsic apoptosis [69].

## 5 Conclusion

In conclusion, both PTX, mesalamine, and their combination effectively protected against experimentally‐induced UC in rats, demonstrating significant anti‐ulcerogenic and colo‐protective effects. These therapeutic benefits are primarily mediated through the enhancement of Nrf2, HO‐1, ZO‐1, and AMPK signaling pathways, alongside the downregulation of NLRP3 expression and suppression of the IL‐6/STAT3 and S1P/SPHK axes. The antioxidant, anti‐inflammatory, and antiapoptotic properties of PTX contribute to its overall protective effects, highlighting its potential as an effective treatment for UC.

## Author Contributions

All authors made a significant contribution to the work.

## Conflicts of Interest

The authors declare no conflicts of interest.

## Data Availability

Data are available upon request.

## References

[1] S. M. El‐Haggar , S. K. Hegazy , M. M. Maher , M. M. Bahgat , and M. M. Bahaa , “Repurposing Metformin as Adjuvant Therapy in Patients With Ulcerative Colitis Treated With Mesalamine: A Randomized Controlled Double‐Blinded Study,” International Immunopharmacology 138 (2024): 112541, 10.1016/j.intimp.2024.112541. 38917525

[2] S. Cucchiara , L. Stronati , and M. Aloi , “Interactions Between Intestinal Microbiota and Innate Immune System in Pediatric Inflammatory Bowel Disease,” Journal of Clinical Gastroenterology 46 (2012): S64–S66. 22955361 10.1097/MCG.0b013e31826a857f

[3] S. Rose‐John , K. Mitsuyama , S. Matsumoto , W. M. Thaiss , and J. Scheller , “Interleukin‐6 Trans‐Signaling and Colonic Cancer Associated With Inflammatory Bowel Disease,” Current Pharmaceutical Design 15, no. 18 (2009): 2095–2103. 19519447 10.2174/138161209788489140

[4] K. Mitsuyama , M. Sata , and S. Rose‐John , “Interleukin‐6 Trans‐Signaling in Inflammatory Bowel Disease,” Cytokine & Growth Factor Reviews 17, no. 6 (2006): 451–461. 17045835 10.1016/j.cytogfr.2006.09.003

[5] M. Coskun , M. Salem , J. Pedersen , and O. H. Nielsen , “Involvement of JAK/STAT Signaling in the Pathogenesis of Inflammatory Bowel Disease,” Pharmacological Research 76 (2013): 1–8. 23827161 10.1016/j.phrs.2013.06.007

[6] X. Han , D. Sosnowska , E. L. Bonkowski , and L. A. Denson , “Growth Hormone Inhibits Signal Transducer and Activator of Transcription 3 Activation and Reduces Disease Activity in Murine Colitis,” Gastroenterology 129, no. 1 (2005): 185–203. 16012947 10.1053/j.gastro.2005.05.018

[7] B. Xia , J. Crusius , S. Meuwissen , and A. Pena , “Inflammatory Bowel Disease: Definition, Epidemiology, Etiologic Aspects, and Immunogenetic Studies,” World Journal of Gastroenterology 4, no. 5 (1998): 446. 11819343 10.3748/wjg.v4.i5.446 PMC4767749

[8] K. Itagaki and C. J. Hauser , “Sphingosine 1‐Phosphate, a Diffusible Calcium Influx Factor Mediating Store‐Operated Calcium Entry,” Journal of Biological Chemistry 278, no. 30 (2003): 27540–27547. 12746430 10.1074/jbc.M301763200 PMC3206310

[9] A. E. Khodir , E. Said , H. Atif , H. A. ElKashef , and H. A. Salem , “Targeting Nrf2/HO‐1 Signaling by Crocin: Role in Attenuation of AA‐Induced Ulcerative Colitis in Rats,” Biomedicine & Pharmacotherapy 110 (2019): 389–399. 30530041 10.1016/j.biopha.2018.11.133

[10] Y. E. Chen , S. J. Xu , Y. Y. Lu , et al., “Asperuloside Suppressing Oxidative Stress and Inflammation in DSS‐Induced Chronic Colitis and RAW 264.7 Macrophages via Nrf2/HO‐1 and NF‐κB Pathways,” Chemico‐Biological Interactions 344 (2021): 109512, 10.1016/j.cbi.2021.109512. 33974900

[11] F. Marín‐Aguilar , L. E. Pavillard , F. Giampieri , P. Bullón , and M. D. Cordero , “Adenosine Monophosphate (AMP)‐Activated Protein Kinase: A New Target for Nutraceutical Compounds,” International Journal of Molecular Sciences 18, no. 2 (2017): 288. 28146060 10.3390/ijms18020288 PMC5343824

[12] N. B. Ruderman , D. Carling , M. Prentki , and J. M. Cacicedo , “AMPK, Insulin Resistance, and the Metabolic Syndrome,” Journal of Clinical Investigation 123, no. 7 (2013): 2764–2772. 23863634 10.1172/JCI67227 PMC3696539

[13] R. A. Saxton and D. M. Sabatini , “mTOR Signaling in Growth, Metabolism, and Disease,” Cell 168, no. 6 (2017): 960–976. 28283069 10.1016/j.cell.2017.02.004 PMC5394987

[14] Q. Li , H. Cheng , Y. Liu , X. Wang , F. He , and L. Tang , “Activation of mTORC1 by LSECtin in Macrophages Directs Intestinal Repair in Inflammatory Bowel Disease,” Cell Death & Disease 11, no. 10 (2020): 918. 33106485 10.1038/s41419-020-03114-4 PMC7589503

[15] S. Saber and E. M. A. El‐Kader , “Novel Complementary Coloprotective Effects of Metformin and MCC950 by Modulating HSP90/NLRP3 Interaction and Inducing Autophagy in Rats,” Inflammopharmacology 29 (2021): 237–251. 32594364 10.1007/s10787-020-00730-6

[16] H. Jiang , Y. Q. Y , W. Jiang , and R. B. Z , “NLRP3 Inflammasome: Activation, Regulation, and Role in Diseases,” SCIENTIA SINICA Vitae 47, no. 1 (2017): 125–131.

[17] F. Yang , Y. Qin , Y. Wang , et al., “Metformin Inhibits the NLRP3 Inflammasome via AMPK/mTOR‐Dependent Effects in Diabetic Cardiomyopathy,” International Journal of Biological Sciences 15, no. 5 (2019): 1010–1019. 31182921 10.7150/ijbs.29680 PMC6535781

[18] F. D'Amico , E. Fasulo , V. Jairath , K. Paridaens , L. Peyrin‐Biroulet , and S. Danese , “Management and Treatment Optimization of Patients With Mild to Moderate Ulcerative Colitis,” Expert Review of Clinical Immunology 20, no. 3 (2024): 277–290. 38059454 10.1080/1744666X.2023.2292768

[19] T. N. Jarada , J. G. Rokne , and R. Alhajj , “A Review of Computational Drug Repositioning: Strategies, Approaches, Opportunities, Challenges, and Directions,” Journal of Cheminformatics 12, no. 1 (2020): 1–23. 33431024 10.1186/s13321-020-00450-7 PMC7374666

[20] S. Pushpakom , F. Iorio , P. A. Eyers , et al., “Drug Repurposing: Progress, Challenges and Recommendations,” Nature Reviews Drug Discovery 18, no. 1 (2019): 41–58. 30310233 10.1038/nrd.2018.168

[21] K. M. Aldossary , L. S. Ali , M. S. Abdallah , et al., “Effect of a High Dose Atorvastatin as Added‐On Therapy on Symptoms and Serum AMPK/NLRP3 Inflammasome and IL‐6/STAT3 Axes in Patients With Major Depressive Disorder: Randomized Controlled Clinical Study,” Frontiers in Pharmacology 15 (2024): 1381523. 38855751 10.3389/fphar.2024.1381523 PMC11157054

[22] S. J. Alarfaj , M. M. Bahaa , T. A. Elmasry , et al., “Fenofibrate as an Adjunct Therapy for Ulcerative Colitis: Targeting Inflammation via SIRT1, NLRP3, and AMPK Pathways: A Randomized Controlled Pilot Study,” Drug Design, Development and Therapy 18 (2024): 5239–5253. 39575188 10.2147/DDDT.S490772 PMC11578921

[23] T. C. Peterson , M. R. Peterson , and J. M. Raoul , “The Effect of Pentoxifylline and Its Metabolite‐1 on Inflammation and Fibrosis in the TNBS Model of Colitis,” European Journal of Pharmacology 662, no. 1–3 (2011): 47–54. 21554874 10.1016/j.ejphar.2011.04.030

[24] H. J. Lee , “Therapeutic Potential of the Combination of Pentoxifylline and Vitamin‐E in Inflammatory Bowel Disease: Inhibition of Intestinal Fibrosis,” Journal of Clinical Medicine 11, no. 16 (2022): 4713. 36012952 10.3390/jcm11164713 PMC9410449

[25] E. Karatay , Ö. G. Utku , H. Erdal , et al., “Pentoxifylline Attenuates Mucosal Damage in an Experimental Model of Rat Colitis by Modulating Tissue Biomarkers of Inflammation, Oxidative Stress, and Fibrosis,” Turkish Journal of Medical Sciences 47, no. 1 (2017): 348–356, 10.3906/sag-1508-98. 28263514

[26] M. M. Bahaa , S. K. Hegazy , M. M. Maher , M. M. Bahgat , and S. M. El‐Haggar , “Pentoxifylline in Patients With Ulcerative Colitis Treated With Mesalamine by Modulation of IL‐6/STAT3, ZO‐1, and S1P Pathways: A Randomized Controlled Double‐Blinded Study,” Inflammopharmacology 32, no. 5 (2024): 3247–3258, 10.1007/s10787-024-01560-6. 39192162

[27] A. E. Khodir , H. Atef , E. Said , H. A. ElKashef , and H. A. Salem , “Implication of Nrf2/HO‐1 Pathway in the Coloprotective Effect of Coenzyme Q10 Against Experimentally Induced Ulcerative Colitis,” Inflammopharmacology 25, no. 1 (2017): 119–135, 10.1007/s10787-016-0305-0. 28050757

[28] N. A. El‐Mahdy , M. E.‐S. El‐Sayad , A. H. El‐Kadem , and S. E.‐S. Abu‐Risha , “Metformin Alleviates Inflammation in Oxazolone Induced Ulcerative Colitis in Rats: Plausible Role of Sphingosine Kinase 1/Sphingosine 1 Phosphate Signaling Pathway,” Immunopharmacology and Immunotoxicology 43, no. 2 (2021): 192–202. 33504231 10.1080/08923973.2021.1878214

[29] S. Murthy , H. S. Cooper , H. Yoshitake , C. Meyer , C. J. Meyer , and N. S. Murthy , “Combination Therapy of Pentoxifylline and TNFα Monoclonal Antibody in Dextran Sulphate‐Induced Mouse Colitis,” Alimentary Pharmacology & Therapeutics 13, no. 2 (1999): 251–260. 10102957 10.1046/j.1365-2036.1999.00457.x

[30] H. A. AlRasheed , S. M. El‐Haggar , S. K. Hegazy , M. M. Maher M. M. Bahgat , and M. M. Bahaa , “Repurposing Atorvastatin, HMGCO‐A Reductase Inhibitor, in Patients with Ulcerative Colitis: A Randomized Controlled Study,” Journal of Clinical Medicine 14, no. 9 (2025): 3077. 40364108 10.3390/jcm14093077 PMC12072543

[31] E. M. El Morsy , R. Kamel , and M. A. Ahmed , “Attenuating Effects of Coenzyme Q10 and Amlodipine in Ulcerative Colitis Model in Rats,” Immunopharmacology and Immunotoxicology 37, no. 3 (2015): 244–251. 25753843 10.3109/08923973.2015.1021357

[32] P. K. Randhawa , K. Singh , N. Singh , and A. S. Jaggi , “A Review on Chemical‐Induced Inflammatory Bowel Disease Models in Rodents,” Korean Journal of Physiology & Pharmacology 18, no. 4 (2014): 279–288. 25177159 10.4196/kjpp.2014.18.4.279 PMC4146629

[33] S. Eğin , M. Ilhan , S. Bademler , et al., “Protective Effects of Pentoxifylline in Small Intestine After Ischemia–Reperfusion,” Journal of International Medical Research 46, no. 10 (2018): 4140–4156. 30027781 10.1177/0300060518786904 PMC6166353

[34] S. Baweja , A. Kumari , P. Negi , et al., “Hepatopulmonary Syndrome Is Associated With Low Sphingosine‐1‐Phosphate Levels and Can Be Ameliorated by the Functional Agonist Fingolimod,” Journal of Hepatology 79 (2023): 167–180. 36996943 10.1016/j.jhep.2023.03.018

[35] O. A. Sukocheva , H. Furuya , M. L. Ng , et al., “Sphingosine Kinase and Sphingosine‐1‐Phosphate Receptor Signaling Pathway in Inflammatory Gastrointestinal Disease and Cancers: A Novel Therapeutic Target,” Pharmacology & Therapeutics 207 (2020): 107464. 31863815 10.1016/j.pharmthera.2019.107464

[36] A. A. Abdin , “Targeting Sphingosine Kinase 1 (SphK1) and Apoptosis by Colon‐Specific Delivery Formula of Resveratrol in Treatment of Experimental Ulcerative Colitis in Rats,” European Journal of Pharmacology 718, no. 1–3 (2013): 145–153. 24055189 10.1016/j.ejphar.2013.08.040

[37] L. W. Maines , L. R. Fitzpatrick , K. J. French , et al., “Suppression of Ulcerative Colitis in Mice by Orally Available Inhibitors of Sphingosine Kinase,” Digestive Diseases and Sciences 53 (2008): 997–1012. 18058233 10.1007/s10620-007-0133-6 PMC2660406

[38] N.‐Y. Kim , H.‐O. Pae , Y.‐C. Kim , et al., “Pentoxifylline Potentiates Nitric Oxide Production in Interleukin‐1β‐Stimulated Vascular Smooth Muscle Cells Through Cyclic AMP‐Dependent Protein Kinase A Pathway,” General Pharmacology: The Vascular System 35, no. 4 (2000): 205–211. 11827727 10.1016/s0306-3623(01)00108-2

[39] S. Murthy , H. Cooper , H. Yoshitake , C. Meyer , C. Meyer , and N. Murthy , “Combination Therapy of Pentoxifylline and TNFalpha Monoclonal Antibody in Dextran Sulphate‐Induced Mouse Colitis,” Alimentary Pharmacology & Therapeutics 13, no. 2 (1999): 251–260. 10102957 10.1046/j.1365-2036.1999.00457.x

[40] S. M. El‐Haggar , S. K. Hegazy , S. M. Abd‐Elsalam , and M. M. Bahaa , “Pentoxifylline, a Nonselective Phosphodiesterase Inhibitor, in Adjunctive Therapy in Patients With Irritable Bowel Syndrome Treated With Mebeverine,” Biomedicine & Pharmacotherapy 145 (2022): 112399, 10.1016/j.biopha.2021.112399. 34775240

[41] Y. Shirakami , T. Kochi , M. Kubota , et al., “Inhibitory Effects of Pentoxifylline on Inflammation‐Related Tumorigenesis in Rat Colon,” Oncotarget 9, no. 74 (2018): 33972–33981. 30338039 10.18632/oncotarget.26119 PMC6188053

[42] A. Goodarzdashti , M. Azadbakht , M. Pourmoradi , and H. Zhaleh , “Effect of Pentoxifylline in the Presence of Staurosporine on Mouse Bone Marrow Mesenchymal Stem Cells,” Cell Journal (Yakhteh) 15 (2013): 42.

[43] M. Z. Kamran and R. P. Gude , “Pentoxifylline Inhibits Melanoma Tumor Growth and Angiogenesis by Targeting STAT3 Signaling Pathway,” Biomedicine & Pharmacotherapy 67, no. 5 (2013): 399–405. 23639230 10.1016/j.biopha.2013.03.020

[44] J. E. Darnell, Jr. , l M. Kerr , and G. R. Stark , “Jak‐STAT Pathways and Transcriptional Activation in Response to IFNs and Other Extracellular Signaling Proteins,” Science 264, no. 5164 (1994): 1415–1421. 8197455 10.1126/science.8197455

[45] H. Dhameliya , V. Thakkar , G. Trivedi , S. Mesara , and R. Subramanian , “Pentoxifylline: An Immunomodulatory Drug for the Treatment of COVID‐19,” Journal of Pure and Applied Microbiology 14, no. 1 (2020): 861–867.

[46] T. W. Costantini , J. Deree , W. Loomis , et al., “Phosphodiesterase Inhibition Attenuates Alterations to the Tight Junction Proteins Occludin and ZO‐1 in Immunostimulated Caco‐2 Intestinal Monolayers,” Life Sciences 84, no. 1–2 (2009): 18–22. 18992758 10.1016/j.lfs.2008.10.007

[47] D.‐y. Xia , H.‐s. Zhang , L.‐y. Wu , X.‐s. Zhang , M.‐l. Zhou , and C.‐h. Hang , “Pentoxifylline Alleviates Early Brain Injury After Experimental Subarachnoid Hemorrhage in Rats: Possibly via Inhibiting TLR 4/NF‐κB Signaling Pathway,” Neurochemical Research 42, no. 4 (2017): 963–974, 10.1007/s11064-016-2129-0. 27933551

[48] N. Sawada , M. Murata , K. Kikuchi , et al., “Tight Junctions and Human Diseases,” Medical Electron Microscopy 36, no. 3 (2003): 147–156. 14505058 10.1007/s00795-003-0219-y

[49] J. Semmler , U. Gebert , T. Eisenhut , et al., “Xanthine Derivatives: Comparison Between Suppression of Tumour Necrosis Factor‐Alpha Production and Inhibition of cAMP Phosphodiesterase Activity,” Immunology 78, no. 4 (1993): 520–525. 8388363 PMC1421886

[50] E. M. Mosalam , A. A. Zidan , E. T. Mehanna , N. M. Mesbah , and D. M. Abo‐Elmatty , “Thymoquinone and Pentoxifylline Enhance the Chemotherapeutic Effect of Cisplatin by Targeting Notch Signaling Pathway in Mice,” Life Sciences 244 (2020): 117299. 31953157 10.1016/j.lfs.2020.117299

[51] C. Windmeier and A. Gressner , “Pharmacological Aspects of Pentoxifylline With Emphasis on Its Inhibitory Actions on Hepatic Fibrogenesis,” General Pharmacology: The Vascular System 29, no. 2 (1997): 181–196. 9251897 10.1016/s0306-3623(96)00314-x

[52] S. Basuroy , A. Seth , B. Elias , A. P. Naren , and R. Rao , “MAPK Interacts With Occludin and Mediates EGF‐Induced Prevention of Tight Junction Disruption by Hydrogen Peroxide,” Biochemical Journal 393, no. 1 (2006): 69–77. 16134968 10.1042/BJ20050959 PMC1383665

[53] J. Deree , J. Martins , T. De Campos , et al., “Pentoxifylline Attenuates Lung Injury and Modulates Transcription Factor Activity in Hemorrhagic Shock,” Journal of Surgical Research 143, no. 1 (2007): 99–108. 17950078 10.1016/j.jss.2007.03.083

[54] T. Y. Ma , G. K. Iwamoto , N. T. Hoa , et al., “TNF‐α‐Induced Increase in Intestinal Epithelial Tight Junction Permeability Requires NF‐κB Activation,” American Journal of Physiology ‐ Gastrointestinal and Liver Physiology 286, no. 3 (2004): G367–G376, 10.1152/ajpgi.00173.2003. 14766535

[55] S. Minguet , M. Huber , L. Rosenkranz , W. W. Schamel , M. Reth , and T. Brummer , “Adenosine and cAMP Are Potent Inhibitors of the NF‐κB Pathway Downstream of Immunoreceptors,” European Journal of Immunology 35, no. 1 (2005): 31–41. 15580656 10.1002/eji.200425524

[56] M. Hekmat , M. F. Schaalan , R. M. Rahmo , D. B. Farag , and L. H. Khedr , “Implications of miRNAs on TGF‐β/TAK1/mTOR Pathway in Mediating the Renoprotective Effects of Pentoxifylline Against Cisplatin‐Induced Nephrotoxicity in Rats,” Toxicology and Applied Pharmacology 404 (2020): 115184. 32777238 10.1016/j.taap.2020.115184

[57] E. Norsted Gregory , A. Delaney , S. AbdelMoaty , et al., “Pentoxifylline and Propentofylline Prevent Proliferation and Activation of the Mammalian Target of Rapamycin and Mitogen Activated Protein Kinase in Cultured Spinal Astrocytes,” Journal of Neuroscience Research 91, no. 2 (2013): 300–312. 23184810 10.1002/jnr.23144

[58] B. M. Hendry , N. Stafford , A. D. Arnold , et al., “Hypothesis: Pentoxifylline Is a Potential Cytokine Modulator Therapeutic in COVID‐19 Patients,” Pharmacology Research & Perspectives 8, no. 4 (2020): e00631. 32715661 10.1002/prp2.631 PMC7383088

[59] X. Li , X. Zhang , Y. Pan , et al., “mTOR Regulates NLRP3 Inflammasome Activation via Reactive Oxygen Species in Murine Lupus,” Acta Biochimica et Biophysica Sinica 50, no. 9 (2018): 888–896. 30060081 10.1093/abbs/gmy088

[60] S. Hamama , M. Gilbert‐Sirieix , M.‐C. Vozenin , and S. Delanian , “Radiation‐Induced Enteropathy: Molecular Basis of Pentoxifylline–Vitamin E Anti‐Fibrotic Effect Involved TGF‐β1 Cascade Inhibition,” Radiotherapy and Oncology 105, no. 3 (2012): 305–312. 23021793 10.1016/j.radonc.2012.08.023

[61] P. A. Welling , Regulation of Renal Potassium Secretion: Molecular Mechanisms. Seminars in Nephrology (Elsevier, 2013). 10.1016/j.semnephrol.2013.04.002 23953799

[62] D. M. Abdallah and H. S. El‐Abhar , “Pentoxifylline Treatment Alleviates Kidney Ischemia/Reperfusion Injury: Novel Involvement of Galectin‐3 and ASK‐1/JNK & ERK1/2/NF‐kB/HMGB‐1 Trajectories,” 2021. 10.1016/j.jphs.2021.03.011 34030796

[63] M. H. Seo , M. Y. Eo , T. T. H. Nguyen , H. J. Yang , and S. M. Kim , “Immunomodulatory Effects of Pentoxifylline: Profiling Data Based on RAW 264.7 Cellular Signaling,” Applied Sciences 11, no. 17 (2021): 8273.

[64] T. O. Khor , M.‐T. Huang , K. H. Kwon , J. Y. Chan , B. S. Reddy , and A.‐N. Kong , “Nrf2‐Deficient Mice Have an Increased Susceptibility to Dextran Sulfate Sodium–Induced Colitis,” Cancer Research 66, no. 24 (2006): 11580–11584. 17178849 10.1158/0008-5472.CAN-06-3562

[65] J. Kim , Y.‐N. Cha , and Y.‐J. Surh , “A Protective Role of Nuclear Factor‐Erythroid 2‐Related Factor‐2 (Nrf2) in Inflammatory Disorders,” Mutation Research: Fundamental and Molecular Mechanisms of Mutagenesis 690, no. 1–2 (2010): 12–23. 19799917 10.1016/j.mrfmmm.2009.09.007

[66] M. Yalniz , U. Demirel , C. Orhan , et al., “Nadroparin Sodium Activates Nrf2/HO‐1 Pathway in Acetic Acid‐Induced Colitis in Rats,” Inflammation 35 (2012): 1213–1221. 22350949 10.1007/s10753-012-9431-z

[67] O. D. Akanji , G. Hassanzadeh , M. Malekzadeh , et al., “Pentoxifylline Promotes Spermatogenesis via Upregulation of the Nrf2‐ARE Signalling Pathway in a Mouse Model of Germ‐Cell Apoptosis Induced by Testicular Torsion–Detorsion,” Reproduction, Fertility and Development 35, no. 7 (2023): 423–432. 37062868 10.1071/RD22168

[68] P. Li , J.‐M. Chen , S.‐H. Ge , et al., “Pentoxifylline Protects Against Cerebral Ischaemia‐Reperfusion Injury Through Ferroptosis Regulation via the Nrf2/SLC7A11/GPX4 Signalling Pathway,” European Journal of Pharmacology 967 (2024): 176402, 10.1016/j.ejphar.2024.176402. 38331339

[69] H. M. El‐Sadek , M. Y. Al‐Shorbagy , M. M. Awny , D. M. Abdallah , and H. S. El‐Abhar , “Pentoxifylline Treatment Alleviates Kidney Ischemia/Reperfusion Injury: Novel Involvement of Galectin‐3 and ASK‐1/JNK & ERK1/2/NF‐κB/HMGB‐1 Trajectories,” Journal of Pharmacological Sciences 146, no. 3 (2021): 136–148. 34030796 10.1016/j.jphs.2021.03.011

